# Targeting Sphingosine-1-Phosphate Signaling in Breast Cancer

**DOI:** 10.3390/ijms25063354

**Published:** 2024-03-15

**Authors:** Masayuki Nagahashi, Yasuo Miyoshi

**Affiliations:** Department of Surgery, Division of Breast and Endocrine Surgery, School of Medicine, Hyogo Medical University, 1-1 Mukogawa-cho, Nishinomiya 663-8501, Hyogo, Japan; ymiyoshi@hyo-med.ac.jp

**Keywords:** sphinegosine-1-phosphate, breast cancer, microenvironment, lymphangiogenesis, lymphatic metastasis, targeted therapy, treatment

## Abstract

In recent years, newly emerging therapies, such as immune checkpoint inhibitors and antibody-drug conjugates, have further improved outcomes for breast cancer patients. However, recurrent and metastatic breast cancer often eventually develops resistance to these drugs, and cure is still rare. As such, the development of new therapies for refractory breast cancer that differ from conventional mechanisms of action is necessary. Sphingosine-1-phosphate (S1P) is a key molecule with a variety of bioactive activities, including involvement in cancer cell proliferation, invasion, and metastasis. S1P also contributes to the formation of the cancer microenvironment by inducing surrounding vascular- and lymph-angiogenesis and regulating the immune system. In this article, we outline the basic mechanism of action of S1P, summarize previous findings on the function of S1P in cancer cells and the cancer microenvironment, and discuss the clinical significance of S1P in breast cancer and the therapeutic potential of targeting S1P signaling.

## 1. Introduction

Breast cancer is the most common cancer among women worldwide, and despite advances in treatment, many women still die from it [1,2]. In recent years, newly emerging therapies, such as immune checkpoint inhibitors and antibody-drug conjugates, have further improved outcomes for breast cancer patients [3,4,5,6,7,8,9,10,11,12]. However, recurrent and metastatic breast cancer often eventually develop resistance to these drugs, and cure is still rare [13,14,15,16,17,18,19]. Further development of new therapies for refractory breast cancer that do not have conventional mechanisms of action is necessary.

Recently, lipid mediators have attracted attention as signaling molecules that play important roles in cancer [20,21,22,23,24,25,26,27]. Among the lipid mediators, sphingosine-1-phosphate (S1P) is a key molecule with a variety of bioactive activities, including those involved in cancer cell proliferation, invasion, and metastasis; S1P has also been shown to contribute to the formation of the cancer microenvironment by inducing surrounding vascular- and lymph-angiogenesis and regulating the immune system [28,29,30,31,32,33,34,35,36]. More than 30 years of research have led to today’s focus on S1P as a factor that controls cellular physiological activity and plays an important role in cancer.

In the first half of the 1990s, Spiegel et al. reported that S1P is a lipid mediator that acts on cells in a way similar to protein growth factors that regulate cell growth [37,38,39,40]. Subsequently, Spiegel et al. and other researchers around the world have clarified the details of S1P-producing enzymes and the specific receptors for S1P, and the wide range of biological activities of S1P have been revealed [41,42,43,44,45,46,47,48,49,50,51,52,53,54,55,56,57,58,59,60]. However, the clinical significance of S1P in cancer patients had not been well understood until recently.

Because S1P is a lipid, it is not easy to accurately quantify its levels, and the significance of S1P in cancer has not been fully elucidated. We have demonstrated that S1P production is upregulated in various cancer types, including breast cancer, and that it contributes to lymphatic metastasis, and is associated with prognosis based on the histological evaluation of phosphorylation of sphingosine kinase type 1 (SphK1), the S1P-producing enzyme [61,62,63,64,65,66,67,68,69]. In this article, we outline the basic mechanism of action of S1P, summarize previous findings on the function of S1P in cancer cells and the cancer microenvironment, and discuss the clinical significance of S1P in breast cancer patients and the therapeutic potential of targeting S1P signaling.

## 2. Molecular Mechanisms of S1P Regulation of Cellular Physiological Functions

**Figure 1:**
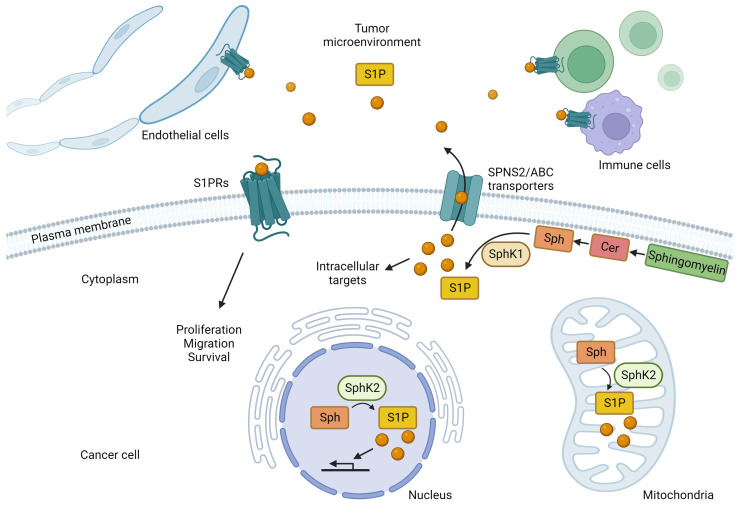
The roles of sphingosine-1-phosphate (S1P) produced by cancer cells. In cancer cells, sphingosine (Sph) produced from ceramide (Cer), one of the components of the cell membrane, is phosphorylated by sphingosine kinases (SphK1 and SphK2) to produce S1P. S1P produced in the cytoplasm by SphK1 is released into the extracellular space by transporters, such as SPNS2 and the ATP-binding cassette (ABC) transporters. Extracellular S1P acts on cancer cells themselves in an autocrine manner and also activates S1P receptors (S1PR) on the cell surface of surrounding cancer cells, immune cells, and endothelial cells in the cancer microenvironment in a paracrine manner, thereby triggering intracellular signals that regulate their physiological functions. SphK1 regulates physiological functions by activating S1P receptors (S1PR) on the cell surface of cancer cells, immune cells, endothelial cells, and other cell types. Stimulation of S1P receptors on cancer cells promotes cancer growth, migration, and survival. S1P produced by SphK2 in the nucleus is thought to contribute to the transcriptional regulation of genes. S1P produced by SphK2 in the mitochondria is also involved in the electron transport system.

Sphingosine kinases produce S1P by phosphorylating sphingosine produced from ceramide, a component of the cell membrane, via the catalytic action of ceramidase [70,71,72,73] (Figure 1). There are two types of sphingosine kinases, SphK1 and SphK2, each with different subcellular localizations [74,75,76,77]; SphK1 is mainly found in the cytoplasm near the plasma membrane, while SphK2 is mainly found in the nucleus and mitochondria [74,75,76,77]. S1P, produced mainly by SphK1 in the cytosol, is released extracellularly by transporters on the plasma membrane and acts as a signaling molecule that regulates cellular functions by acting on S1P-specific G protein-coupled receptors on the cell surface via autocrine and paracrine modes of action [78,79,80,81,82]. ATP-binding cassette (ABC) transporters, such as ABCC1 and ABCG2, and the S1P-specific transporter, Spns2, transport intracellular S1P to the cell exterior [82,83,84,85,86,87,88,89]. Extracellularly released S1P stimulates one of five S1P receptors (S1PR1-5) that exhibit tissue-specific expression patterns and each S1P receptor binds to a different G protein and regulates a wide range of downstream signaling pathways and numerous biological processes [90,91,92,93,94,95,96,97,98,99,100,101,102,103,104,105,106,107,108]. The intracellular production of S1P, followed by its release and extracellular action, is called “inside-out” signaling and is characteristic of S1P signaling [109,110].

In addition to “inside-out” signaling, S1P also acts directly via intracellular targets. For example, tumor necrosis factor-α (TNF-α) and interleukin-1 (IL-1) increase the levels of intracellular S1P that binds directly to TNF-α receptor-associated factor 2 by activating SphK1 [111]. TNF-α receptor-associated factor 2 is an important regulator of nuclear factor-κB signaling and cellular apoptosis suppression 2, and plays an important role in the regulation of apoptosis. In addition, it acts via lysine-63 binding polyubiquitination to enhance E3 ubiquitin ligase activity [111]. SphK2 exists in the nucleus and mitochondria and plays important roles in these organelles (Figure 1). S1P produced by SphK2 in the nucleus specifically binds to histone deacetylase (HDAC) 1 and HDAC2, inhibiting their enzymatic activity and preventing the removal of acetyl groups from lysine residues within histone tails [112]. As a result, S1P acts as an HDAC inhibitor that is involved in the transcriptional regulation of genes, and it plays a role in higher-order functional regulation in the brain [112,113]. We also reported that SphK2 and S1P regulate the transcription of genes encoding enzymes involved in metabolism in the liver [114,115,116,117,118]. In mitochondria, it has been suggested that SphK2 and S1P may be involved in energy metabolism via the electron transfer system [119]. In brief, S1P, mainly produced by SphK2 in mitochondria, binds to prohibitin 2, which plays an important role in regulating cytochrome-c oxidase assembly and mitochondrial respiration [119].

The concentration levels of S1P have been found to be very tightly regulated by the balance between its synthesis and degradation. S1P is converted to sphingosine by two specific S1P phosphatases (SPP1 and SPP2) belonging to the magnesium-dependent, N-ethylmaleimide-insensitive type 2 lipid phosphate phosphohydrolase family in the endoplasmic reticulum, where phosphate groups are removed [120,121]. S1P is also irreversibly degraded to hexadecenal and phosphoethanolamine by pyridoxal phosphate-dependent S1P lyase [122,123]. Phosphoethanolamine is subsequently recycled for phosphatidylethanolamine biosynthesis [124].

In the human body, the blood concentration of S1P is finely regulated to keep it at the relatively high level of 1–2 µM [125]. In mice, the half-life of S1P in plasma has been reported to be about 15 min, suggesting rapid clearance by enzymes, such as S1P phosphatase and S1P lyase, or the rapid uptake of S1P into cells [126,127,128]. This rapid turnover of S1P in the blood suggests the presence of a large number of S1P-supplying cells that are involved in maintaining high S1P levels in the blood [128]. The cells responsible for S1P synthesis and secretion into the blood include erythrocytes, endothelial cells, platelets, macrophages, and mast cells [129,130,131,132,133,134].

Compared to the S1P concentration in the blood, the concentration in lymphatic fluid has rarely been measured. The difficulty in collecting lymphatic fluid from clinical samples, and even in animal experiments, may be the reason for the paucity of data. We collected lymphatic fluid from the cisterna chyli of mice using filter paper, extracted S1P from it and quantified its concentration using mass spectrometry. S1P concentrations in lymphatic fluid were in the range of 0.1 to 0.3 μM, while those in plasma were greater than 0.5 μM [135]. The levels of S1P in lymphatic fluid were in agreement with previous reports from other groups [136,137]. We confirmed that the concentration of S1P in lymphatic fluid was significantly higher than that in normal tissue, such as mesenteric lymph nodes [135]. The main source of S1P in the lymphatic fluid is thought to be produced by SphK1 in lymphatic endothelial cells, which is thought to be released into the lymphatic fluid by the Spns2 transporter [138].

In peripheral tissues, S1P concentrations are maintained in the lower range of several nM to several tens of nM by the action of S1P-degrading enzymes, such as S1P phosphatases and S1P lyases [139]. This concentration gradient of S1P among the blood, lymph, and peripheral tissues plays an essential role in regulating immune cell trafficking [138]. In Spns2-deficient mice, S1P concentrations are increased in several specific tissues, including lymph nodes, interstitial fluid, and lymph fluid, suggesting that Spns2 deficiency causes dysregulation of the S1P concentration gradient [140]. As a result, the lymphatic vascular network is disrupted, and the number of lymphocytes in the lymph nodes is reduced in Spns2-deficient mice [140].

S1P is important in the egress of lymphocytes from the lymphoid organs, including the thymus and lymph nodes [141,142]. S1P is considered a circulation marker that allows immune cells to find blood and lymphatic vessels and stabilizes the vascular system by acting on endothelial cells [143]. S1PR1 and sphingosine kinases have an important role in the maturation of vascular and lymphatic vessels [143,144,145,146]. T-cells develop in the thymus, a primary lymphoid organ and S1PR1 signaling is essential for T-cells to exit the thymus after maturation; when S1PR1 is lost in T-cells, mature T-cells outside the thymus are almost completely lost, but mature T-cells within the thymus accumulate [147].

After maturation, lymphocytes circulate through secondary lymphoid organs, such as the lymph nodes, spleen, and Peyer’s patches, to encounter antigens [138,148]. In vivo imaging of S1PR1-deficient cells and control T-cells exiting the lymph nodes suggest that the primary role of S1PR1 is to allow cells to cross the endothelial barrier; both S1PR1-deficient cells and control cells reach the endothelial intercellular spaces in the cortical lymph sinuses and extend their projections, but control T-cells migrate 10 times more frequently than S1PR1-deficient cells [149,150]. In T-cells, S1PR1-mediated exit signals compete with retention signals from chemokine receptor 7 and C-X-C chemokine receptor type 4 [151,152].

S1P is a lipid chemoattractant that directs cells not only from lymphoid tissues but also non-lymphoid tissue with relatively low S1P concentrations to circulating fluids with relatively high S1P concentrations [153,154,155,156]. The role of S1P signaling in T-cell egress from non-lymphoid tissues has been well studied in memory T-cells [141]. Memory T-cells remain at the site of infection and provide strong protection against reinfection. Transcriptional analysis of memory T-cells has revealed that S1pr1 is strongly downregulated in these cells [157,158,159]. Recent experiments further support the hypothesis that S1P levels increase in inflammation, prolonging T-cell residence time in the location of inflammation [160].

## 3. S1P in Cancer Cells and the Interaction between Cancer and the Microenvironment

Cancer cells often have abundant SphK1 in the cytoplasm, and S1P production is enhanced [161,162]. In cancer development and progression, S1P “inside-out” signaling plays a critical role, and S1P acts on S1P receptors on the plasma membrane of the cancer cell itself and in the surrounding microenvironment via autocrine and paracrine modes of action [29,163]. The expression of S1PR1 and S1PR3 is often higher in breast cancer, suggesting that signaling through these receptors contributes to cancer growth and invasion [164,165,166,167,168,169]. S1P also acts on S1PR1 on vascular endothelial cells to induce angiogenesis [170,171] and on S1PR1 on immune cells to promote migration [172]. Thus, the SphKs/S1P/S1PRs signaling pathway is thought to contribute to the formation of the cancer immune microenvironment [34,173].

The cancer microenvironment is composed of host tissues, including blood vessels, lymphatic vessels, immune cells, stromal cells, extracellular matrix, and the stromal fluid that fills this space [174,175,176,177]. The interaction between cancer cells and their microenvironment is important during cancer development and progression, and it is essential to understand the molecular mechanisms involved in this interaction [174,175,176,177,178,179]. Cancer cells are thought to secrete bioactive molecules, such as cytokines, chemokines, and lipid mediators, into the microenvironment to influence cancer progression [179]. On the other hand, noncancerous cells in the microenvironment also release bioactive molecules that influence cancer cells. S1P regulates the development and progression of cancer by promoting cell proliferation, migration, angiogenesis, and lymphangiogenesis [170,179]. The effects of S1P on the interaction between cancer and the microenvironment include the following: (1) S1P produced by cancer cells acts on cancer cells themselves to promote proliferation, migration, and viability; (2) S1P produced by cancer cells acts on the microenvironment to induce vascular and lymphangiogenesis, immune responses, chronic inflammation, and stromal reactions; and (3) cytokines, such as IL-6 and TNF-α, produced by cancer cells act on stromal cells in the microenvironment, and the stromal cells produce S1P and affect the cancer cells [32,179,180].

In mice transplanted with the 4T1 breast cancer cell line, S1P levels in the tumor gradually increase with tumor growth, reaching twice the level of S1P in the normal mammary gland, and serum S1P levels increase significantly, from 800 pmol/mL to about 1200 pmol/mL [181]. SK1-I, an inhibitor of SphK1, reduces S1P levels in tumors to levels comparable to those in the mammary glands of mice without transplanted tumors, and serum S1P levels are also reduced to levels comparable to those in mice without transplanted tumors [181]. Furthermore, stage IIIA breast cancer patients with lymph node metastases have a two-fold increase in serum S1P concentrations compared to age– and ethnicity-matched healthy volunteers [181]. Taken together, it appears that S1P produced by the tumor not only affects the S1P concentration in the tumor but also affects the S1P concentration in the systemic circulation, which is related to cancer progression.

Tissue interstitial fluid surrounds cells in the microenvironment and is drained into lymphatic vessels to become lymph fluid. It eventually enters the veins and joins the systemic circulation. In normal tissues, S1P in tissue fluid is maintained at a low concentration by the aforementioned S1P-degrading enzyme. When S1P is measured after collection by low-speed centrifugation, the concentration in the interstitial fluid of human breast cancer tissue is about several hundred picomoles, approximately three times higher than in the interstitial fluid of normal human mammary glands [139]. This suggests that the S1P concentration in cancer tissue interstitial fluid is increased by the enhanced production and release of S1P in cancer cells and that higher concentrations of S1P flow into lymph vessels. The possibility that S1P released from cancer cells acts on lymphatic endothelial cells and promotes metastasis of cancer cells has been confirmed in animal experiments [181].

Angiogenesis brings oxygen and nutrients to cancer cells as new blood vessels extend into the cancer, provides a conduit for cancer cells to metastasize, and regulates the rate of cancer growth and progression [171,182]. Neutralization of extracellular S1P by anti-S1P antibodies has an inhibitory effect on angiogenesis, tumor growth, and metastasis in animal models, indicating that extracellular S1P regulates angiogenesis in cancer [183]. A study reported that the addition of 1 mg/mL of anti-S1P monoclonal antibody to the medium inhibits the S1P-induced migration of human umbilical vein endothelial cells by 70% [183]. The study also evaluated the ability of S1P to promote vascular endothelial cell infiltration into Matrigel plugs in vivo and found that animals implanted with Matrigel containing 5 mM S1P have a significant, 6-fold increase in vascular endothelial cell density compared to those implanted with Matrigel lacking S1P [183]. Treatment with an anti-S1P monoclonal antibody also suppresses vascular endothelial cell infiltration to a level comparable to antibody-treated animals and controls that did not receive S1P [183]. Data from mouse models and human patient samples suggest that SphK1-upregulated tumors themselves may be an important source of S1P [181,184,185,186]. More importantly, endothelial cells have also been found to synthesize and release S1P [127,187,188,189].

S1P signaling also plays an important role in lymphangiogenesis [170,190,191]. Specifically, S1P induces lymphatic endothelial cell tube formation in an S1PR1-dependent manner, and S1P is also involved in cancer-induced lymphangiogenesis [181,192,193]. Lymphangiogenesis induced by angiopoietin-2 is suppressed by SphK1-specific pharmacological inhibitors, suggesting that there is cross-talk between angiopoietin-2 and S1P signaling pathways and that both interactively contribute to lymphangiogenesis [170,181]. The lymphatic endothelial cell-specific deletion of SphK1 in SphK2 knockout mice results in a complete loss of S1P production in lymphatic vessel endothelial cells, thereby inhibiting lymphatic vessel maturation, suggesting that SphKs and S1P are required for proper development of lymphatic vessel endothelial cells [194].

S1P is also associated with inflammation in the interaction between cancer and the microenvironment [34,195,196]. We have shown that S1P is involved in chronic intestinal inflammation and colitis-associated cancer in a mouse model of inflammatory carcinogenesis [197]; that is, S1P contributes to the production of IL-6, which is regulated by nuclear factor kappa B and to the constant activation of the transcription factor, signal transducer and activator of transcription 3 (STAT3), resulting in the upregulation of S1PR1, and consequently the high expression of S1PR1 [197,198]. In addition, epidemiological data have shown that obesity is a risk factor for postmenopausal breast cancer and exacerbates cancer progression, and the upregulation of S1P/S1PR1/STAT3 signaling has also been implicated in a mouse model of breast cancer associated with obesity [196]. This suggests that S1P plays a key role in the maintenance of chronic inflammation and cancer progression in obesity-associated breast cancer.

Since S1P is strongly involved in immune cell migration, it is assumed to play a significant role in the formation of the tumor immune microenvironment [199]. Memory T-cells in tumor tissue have been reported to be characterized by the absence of S1PR1 [200], which may suggest that memory T-cells are able to remain in the location of tumor tissue for long periods of time by not being receptive to S1P signaling via S1PR1. On the other hand, for regulatory T-cells (Tregs), Stat3-mediated S1PR1 signaling has been shown to be important for Treg migration to tumors [201]. One study showed that increased S1PR1 in CD4+ T-cells promotes STAT3 activation and JAK/STAT3-dependent Treg tumor migration, while ablation of STAT3 in T-cells reduces tumor-associated Treg accumulation and tumor migration [202]. These results demonstrate the importance of S1PR1 signaling in peripheral and tumor Treg cells. In our study, which analyzed the association between immune-related genes and Sphk1 gene expression in breast cancer using the Cancer Genome Atlas database, breast cancers with high Sphk1 expression are associated with increased expression of immune-related genes, such as CD68, CD163, CD4, and forkhead box protein 3, which are associated with increased myeloid-derived suppressor cells (MDSCs), and Treg infiltration is suggested to be increased [203]. Based on previous findings, S1P signaling appears to act in the direction of promoting the migration of immune cells that suppress tumor immunity in the tumor immune microenvironment. The function of S1P in tumor immunity, including its association with immune checkpoint inhibitors, is intriguing and deserves further study.

## 4. Clinical Significance of S1P in Breast Cancer Patients

**Figure 2:**
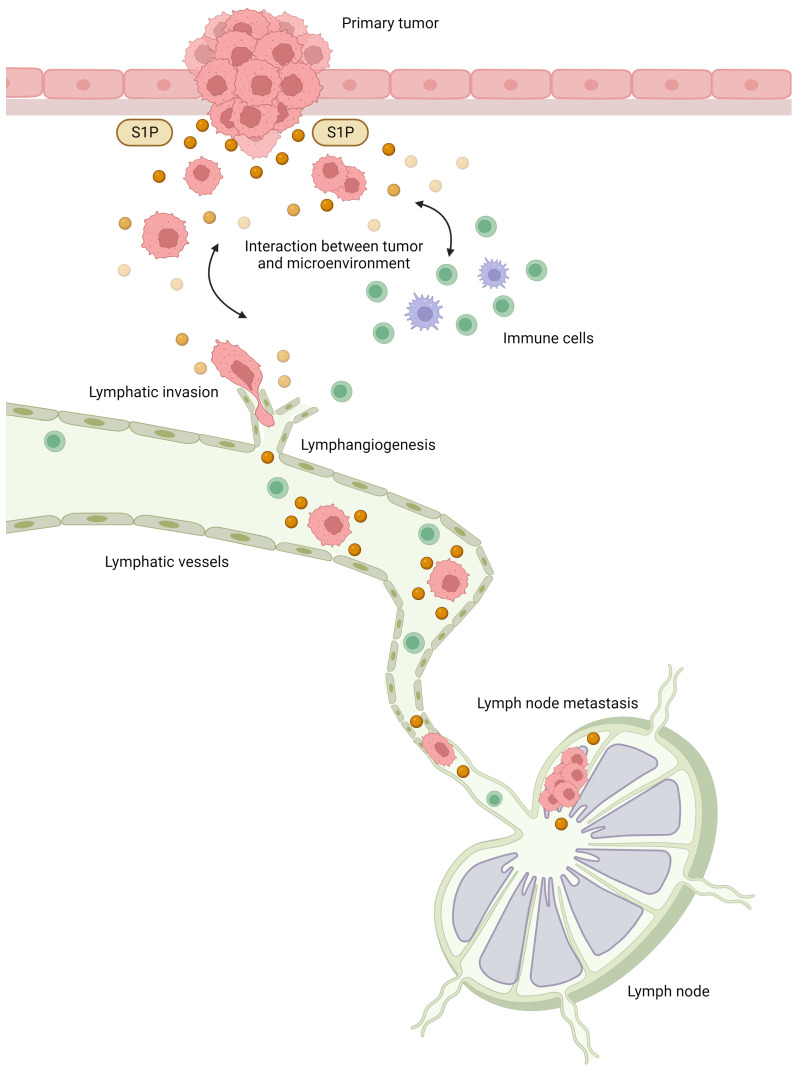
The roles of sphingosine-1-phosphate (S1P) in lymphatic metastasis. S1P produced by cancer cells is released into the interstitial fluid and contributes to the interaction between cancer and the microenvironment, together with cytokines and chemokines. In the cancer microenvironment, S1P promotes cancer cell proliferation and invasion and contributes to the formation of a favorable microenvironment for cancer progression through angiogenesis, lymphangiogenesis, and immune cell mobilization. S1P may contribute to lymphangiogenesis by acting on lymphatic endothelial cells, and S1P in lymphatic vessels may also contribute to the survival of free-floating cancer cells and lymph node metastasis.

Our previous studies have revealed various roles for S1P in the interaction between cancer and the microenvironment [34], and we speculated that S1P may play a major role in lymphatic metastasis (Figure 2). In brief, S1P produced by cancer is released into the interstitial fluid and contributes to the interaction between cancer cells and the microenvironment, together with cytokines and chemokines, which also regulate the physiological functions of cancer cells and mesenchymal cells in the cancer microenvironment. In the cancer microenvironment, S1P promotes cancer cell proliferation and invasion and contributes to the formation of a favorable microenvironment for cancer progression via angiogenesis, lymphangiogenesis, and immune cell mobilization. S1P may contribute to lymphangiogenesis by acting on lymphatic endothelial cells, and S1P in lymphatic vessels may also contribute to the survival of free-floating cancer cells and lymph node metastasis based on our hypothesis.

S1P is expected to be associated with clinical outcomes because it affects the tumor microenvironment and is involved in the developmental progression of cancer. Indeed, previous studies have reported that S1P and SphK1 are associated with outcomes in breast cancer patients [204,205,206]. High SphK1 expression by microarray analysis was associated with significantly worse disease-free survival in combined datasets of 968 breast cancer patients [204]. It had been expected that the high expression levels of SphK1 might result in high levels of S1P in the cancer tissue. We previously confirmed that patients with positive-expression of phosphorylated SphK1 (pSphK1) on breast cancer specimens do indeed show significantly higher levels of S1P in the breast cancer tissue as determined using mass spectrometry [65]. Mass spectrometry analysis of frozen cancer tissue samples in many cancer types also shows significantly higher concentrations of S1P in cancer tissues compared to normal tissues [61,62,63,64]. Regarding breast cancer, our analysis using mass spectrometry shows that S1P concentrations are approximately twice as high in breast cancer as in normal mammary glands [61]. Interestingly, S1P levels in the breast cancer tissue measured using mass spectrometry in patients with lymph node metastasis are significantly higher than those in patients with negative nodes [65].

Immunostaining for pSphK1 is much cheaper and simpler to perform than mass spectrometry and can be analyzed using paraffin blocks rather than fresh frozen samples, making it suitable for studying a larger number of clinical cases. We performed immunostaining using pSphK1 antibodies on surgical specimens from cancer patients and found that pSphK1 is overexpressed in many types of cancer, including breast, gastric, esophageal, pancreatic, biliary, and liver cancers [62,63,64,65,66,67,68,69]. We previously examined 275 breast cancer patients utilizing pSphK1 immunostaining, and the association between pSphK1 expression and clinicopathological factors was analyzed [206]. pSphK1 positivity was about 20% in patients without lymph node metastasis, whereas pSphK1 positivity was about 40% in patients with lymph node metastasis, showing a significant association of the presence of lymph node metastasis with the expression of pSphK1. Furthermore, the clinical stage was also correlated with pSphK1 expression; pSphK1 positivity was approximately 20% in stage I breast cancer but was significantly increased to 60% in stage III cases. Furthermore, survival analysis of those patients revealed that patients with breast cancers that express both pSphK1 and ABCC1 have significantly shorter disease-free survival compared to the others [206]. It was also revealed that increased S1P production, suggested by higher expression of pSphK1, is associated with lymphatic metastasis in various cancer types other than breast cancer, supporting the hypothesis based on previous basic experimental data [62,63,64,65,66,67,68,69].

Breast cancer is treated based on subtype defined by hormone receptors and human epidermal growth factor receptor 2 (HER2) receptor expression; pharmacological inhibition of 17β-estradiol (E2) production or binding of E2 to estrogen receptor (ER) is an effective treatment for patients with ER-positive breast cancer, and ER status is an important prognostic factor [207]. We previously reported that there is an S1P-mediated pathway in E2 signaling [82]. The binding of E2 to ER stimulates the release of S1P via the ABC transporters, ABCC1 and ABCG2, and the released S1P binds to and activates S1P receptors [82]. Activation of S1P receptors promotes breast cancer growth, progression, and invasion by stimulating downstream ERK1/2 [82]. Thus, S1P may contribute to the non-genomic signaling of E2 at the plasma membrane of cells, where they can propagate signal transduction through kinase pathways, such as AKT and MAPK pathways [82,208]. In ER-positive breast cancer, high expression of SphK1 has been reported to be associated with poor prognosis, and SphK1 has been shown to be associated with the development of tamoxifen-resistant early recurrence during tamoxifen treatment [164,209].

HER2 overexpression is a major determinant of breast cancer progression, and S1P signaling may contribute to this; S1PR4 stimulates the ERK1/2 pathway in ER-negative HER2-positive MDA-MB-453 breast cancer cells through a HER2-dependent mechanism [210]. S1PR4 and high expression of SphK1 are associated with shorter survival in breast cancer patients, indicating the importance of S1PR4 and SphK1 in the progression of breast cancer [205]. Interestingly, for S1P concentrations in breast cancer tissues, we found that stronger HER2 expression was associated with lower S1P concentrations [65]. Since both HER2 and SphK1 are strong activators of survival signaling pathways, such as the MAPK pathway, and the HER2 signaling is highly autonomous, it is possible that negative feedback from HER2 signaling suppresses SphK1 activation in HER2-positive breast cancer. However, further studies are needed to clarify the relationship between S1P signaling and HER2 expression.

Triple-negative breast cancer has a particularly poor prognosis because it is often biologically more malignant than other subtypes and has limited treatment options [211]. LM2-4 cells, which have acquired a lung metastasis phenotype from the triple-negative cell line, MDA-MB-231, require SphKs/S1P/S1PRs signaling for their growth, survival, and cell motility [212]. PF-543, a selective and potent inhibitor of SphK1, does not inhibit proliferative signaling in parental MDA-MB-231 cells but inhibits the proliferative signaling of AKT, ERK, and p38 MAP kinase pathways in LM2-4 cells [212]. These observations suggest a contribution of SphKs/S1P/S1PRs signaling in metastatic triple-negative breast cancer and that S1P signaling may be a therapeutic target in triple-negative breast cancer [213].

Although S1P levels in cancer tissue have been shown to correlate with lymphatic metastasis and patient prognosis, the clinical significance of S1P levels in the blood of cancer patients has been less well understood. We investigated ER-positive HER2-negative breast cancer and quantified plasma S1P in patients using mass spectrometry and compared S1P levels with clinicopathological factors [214]. We found that higher plasma S1P levels are associated with larger tumor size, positive lymph nodes, and advanced-stage cancer [214]. Considering that ER-positive HER2-negative breast cancer patients have particularly high S1P levels in cancer tissues compared with other subtypes and that previous basic studies have shown that “inside-out” signaling of S1P contributes to the signaling of the ER non-genomic pathway, S1P is predicted to play an important role in ER-positive breast cancer in combination with estrogen signaling [82]. Therefore, the fact that the S1P concentration in the peripheral blood of patients with ER-positive breast cancer is associated with the degree of cancer progression suggests that the function of S1P in cancer linked to estrogen signaling may be reflected in changes in the peripheral blood.

As mentioned above, animal studies have suggested that S1P links cancer and inflammation, but translational research using clinical specimens has also confirmed this cancer-inflammation relationship [67]. Serum S1P levels are found to be significantly increased in breast cancer patients with obesity [196]. This finding may be a corollary to the involvement of the S1P/S1PR1/STAT3 signaling in breast cancer patients with obesity, similar to the results of animal studies. S1P produced by cancer cells and released into the microenvironment may act on immune cells to promote cancer development and progression. According to the results of the Cancer Genome Atlas database analysis, increased expression of sphk1 in HER2-negative breast cancer tissue is associated with increased expression of immune-related molecules, such as TNF-α, IL-6, and transforming growth factor-β, and probably immune cells that suppress tumor immunity, such as Tregs and MDSCs [203]. Further clarification of the mechanism of interaction between S1P and the suppressive immune cells, Tregs, and MDSCs, and their therapeutic applications are expected in the future.

## 5. Targeting S1P as Therapy for Advanced Breast Cancer

In addition to treatment with anti-S1P antibodies targeting S1P itself [183,215], treatment with inhibitors of S1P receptors and S1P-producing enzymes is being considered to target the S1P signaling pathway [207], and new agents are being developed. FTY720 (fingolimod), the first molecularly targeted drug approved for multiple sclerosis, has been shown to inhibit S1P receptors other than S1PR2 and also inhibit SphK1 in basic studies [216,217,218,219]. Thus, by inhibiting S1P signaling, FTY720 has been shown to suppress the growth and metastasis of various cancers in cellular and animal studies [220,221,222,223]. Because FTY720 has the side effect of immunosuppression, which lowers the number of lymphocytes in the blood, its clinical application as an anticancer drug has not progressed, but efforts are being made to develop new drugs to overcome these side effects [224].

Technological advances have also led to the development of new S1P signaling inhibitors [225]. The function of S1P in nuclear gene regulation may also play an important role in cancer and is a subject for future research [77]. Antibody drugs against S1P have also been developed and are currently in clinical development [183,215]. A number of molecularly targeted drugs that act more specifically on S1P receptors have been developed and are being tested in clinical trials as potential treatments for multiple sclerosis and inflammatory bowel disease [226,227,228,229].

Technological advances have also led to the development of new S1P signaling inhibitors. The function of S1P in nuclear gene regulation may also play an important role in cancer and is a subject for future research [40]. Antibody drugs against S1P have also been developed and are currently in clinical development [103,129]. A number of molecularly targeted drugs that act more specifically on S1P receptors have been developed and are being tested in clinical trials as potential treatments for multiple sclerosis and inflammatory bowel disease [138,139,140,141].

Since the importance of S1P in cancer varies depending on the cancer type, subtype, degree of progression, presence or absence of metastases and the organs to which it spreads, and the cancer microenvironment, clinical application of therapies targeting S1P signaling will require identifying the patient population for whom the therapy will be most effective [230,231]. Therefore, the development of therapies targeting S1P signaling requires the selection of target patient groups, the development of S1P signaling inhibitors, and consideration of combination therapy with anti-cancer drugs and molecular-targeted drugs. A variety of agents have been developed to regulate S1P signaling, each with different targets, and clinical development strategies must be tailored to the pathophysiology of the cancer [181,226,227,228,229,232,233,234,235,236,237,238,239,240,241,242,243,244,245,246,247]. S1P signaling inhibitors developed to date, which could be applied to cancer therapy, are listed in Table 1. Since S1P contributes to inflammation-based carcinogenesis, as in the case of colorectal cancer carcinogenesis from ulcerative colitis, suppression of S1P signaling may be expected to have a preventive effect on carcinogenesis. However, chemoprevention of cancer using drugs would be a later priority in its development from a safety perspective. More realistically, clinical development of the drugs listed in Table 1 as single agents or in combination with existing therapies for the treatment of advanced cancer with metastasis is desirable.

Therapy targeting S1P is thought to affect the microenvironment while suppressing the cancer itself. In particular, it may be effective in inflammation-associated cancers because of its strong anti-inflammatory effect [248,249,250]. In the field of breast cancer, it may be effective, especially in breast cancer associated with obesity and inflammatory breast cancer. Moreover, FTY720, Ozanimod, Siponimod, and Ponesimod are in clinical use for the treatment of multiple sclerosis, meaning that these drugs are effective in crossing the blood–brain barrier. Its potential as a therapeutic agent for brain metastases appears to be promising. Considering the significant impact of S1P on tumor immunity, further studies are needed, but there is great promise for the future use of S1P signaling modulation in immunotherapy.

## 6. Conclusions

In this article, the basic mechanism of action of S1P, a lipid mediator, the function of S1P in cancer cells and the cancer microenvironment based on in vitro and in vivo experiments, and the clinical significance of S1P based on clinical studies are introduced, and its potential for therapeutic application is discussed. Advances in science and technology have made it possible to quantify S1P, which was previously difficult, and to elucidate its clinical significance. Further technological innovations may enable the clinical application of S1P as a biomarker and therapeutic target, and we look forward to the development of future translational research.

## Figures and Tables

**Table 1:** List of major drugs targeting S1P signaling that could be applied to cancer therapy.

Drug	Target	Indications/Observations	Phase of Clinical Use	References
FTY720	SphK1, S1PR1, 3-5	Multiple sclerosis	FDA-approved	[232,233]
SK1-I	SphK1	Leukemia, Breast cancer, etc.	Pre-clinical	[181,234,235]
PF543	SphK1	Colorectal, prostate, and ovarian cancer, etc.	Pre-clinical	[236,237,238,239,240]
SKI-II	SphK1	Colon cancer	Pre-clinical	[241]
SK-F	SphK1	Breast cancer	Pre-clinical	[242]
ABC294640	SphK2	Multiple myeloma, Advanced solid tumors	Phase I	[243,244]
MP-A08	SphK1 and SphK2	Lung adenocarcinoma, Acute myeloid leukemia	Pre-clinical	[245]
Amgen 82	SphK1 and SphK2	Reduction in plasma S1P in nude mice	Pre-clinical	[246]
Ozanimod	S1PR1	Ulcerative colitis, Multiple sclerosis	FDA-approved	[226,227]
Sponimod	S1PR1 and S1PR5	Multiple sclerosis	FDA-approved	[228]
Ponesimod	S1PR1	Multiple sclerosis	FDA-approved	[229]
Sonepcizumab	S1P	Renal cell carcinoma	Phase II	[247]

## Data Availability

No new data were created in this study.
